# Teff Yield and Nutrient Uptake Partitioning During Prolonged Waterlogging: A Preliminary Greenhouse Study

**DOI:** 10.1002/pei3.70043

**Published:** 2025-03-10

**Authors:** Mulugeta Demiss, Upendra Singh, Job Fugice, Zachary P. Stewart, Latha Nagarajan

**Affiliations:** ^1^ Research Division International Fertilizer Development Center (IFDC) Muscle Shoals Alabama USA; ^2^ Center for Agriculture‐Led Growth, Bureau for Resilience and Food Security United States Agency for International Development (USAID) Washington DC USA; ^3^ SOILS Consortium Coordination Office International Fertilizer Development Center (IFDC) Washington DC USA

**Keywords:** aerenchyma, Ethiopia, Quncho, tolerance, transplanting, Vertisols

## Abstract

There is a lack of studies examining the effects of prolonged waterlogging on both yield and nutrient uptake partitioning in teff. A greenhouse study was conducted to assess the impact of different durations of waterlogging on teff's growth, yield, nutrient uptake and partitioning among grain, straw, and root components. Teff plants were subjected to five waterlogging durations as days after transplanting (DAT) ranging from upland to waterlogging to the entire growing period (WHOLE). No significant differences among treatments for most of the study parameters were observed. However, both grain and straw dry matter yields showed an inconsistent trend with the waterlogging duration. On the other hand, the WHOLE treatment resulted in significantly greater root weight compared to the control treatment. Nitrogen (N), phosphorus (P), and potassium (K) uptakes were reduced in grain and straw but increased in roots as the duration of waterlogging increased. Overall, the results suggest that teff can tolerate waterlogging conditions throughout the entire growing period. These results are discussed, and the need for further studies to understand the regulation of root metabolism and physiological mechanisms responsible for teff's tolerance to prolonged waterlogging is stressed.

## 1 Introduction

Teff [ *Eragrostis tef* (Zucc.) Trotter] is an indigenous staple cereal crop of Ethiopia, where it originated and diversified (Assefa et al. 2011). It belongs to the Poaceae family and to the Chloridoideae subfamily (Assefa et al. 2015). Depending on variety and altitude, teff requires 90–130 days for growth (Gebretsadik et al. 2009). Teff is a warm‐season annual grass that has rapid seed germination and seedling development. It is also well adapted to dry climates, soil, and moisture regimes. Teff responds to fertilization, particularly the application of balanced fertilization, containing potassium in Vertisols, which improves yields (Habte and Boke 2017; Misskire et al. 2019; Mulugeta et al. 2020). Teff is becoming popular in different parts of the world recently due to its beneficial effect on human health as a gluten‐free crop (Hopman et al. 2008; Miller 2010) and a rich source of protein and nutrients (Ashenafi 2006; Bultosa 2007). Teff's high nutritional profile, such as its high dietary fiber, thiamine, phosphorus (P), calcium (Ca), iron (Fe), and copper (Cu) contents, and an excellent composition of amino acids essential for humans (Doris 2002; Abebe et al. 2007; Piccinin et al. 2010), makes it an attractive crop worldwide. The majority of teff production is conducted in Ethiopia, but it is widely grown in Australia, China, India, South Africa, the USA, the Netherlands, and Germany, South Africa being the major exporter of teff for the European market. It is attracting the attention of growers because of its growing demand and popularity as palatable forage and high‐quality food.

Teff is a highly versatile crop with respect to adaptation to different agroecologies. It covers the largest area annually in Ethiopia at 23% of its arable land and ranks second in grain production (16.12%) in the 2019/20 cropping season. An area of 3.0 million hectares was cultivated and yielded 5.5 million metric tons (Central Statistics Agency of Ethiopia, 020).

Waterlogging is one of the primary abiotic stresses that adversely hampers crop growth and yields. It disrupts gas exchange between roots and the atmosphere, leading to a rapid decline in oxygen levels in the root zone while increasing carbon dioxide and ethylene concentrations (Setter and Waters 2003). Oxygen deficiency in waterlogged soils impairs root respiration, reducing water and nutrient uptake due to decreased root conductivity. This condition diminishes N uptake by plants and can cause wilting under warm conditions due to high transpiration rates. Additionally, excessive soil moisture contributes to increased nutrient losses (Kaur et al. 2020). Studies estimate that waterlogging stress affects around 10% of global cropland, with yield reductions ranging from 15% to 80%, depending on genotype, environmental conditions, growth stage, and the duration of waterlogging (Mancuso and Shabala 2010; Li et al. 2011; Prasanna and Ramarao 2014).

The impact of waterlogging on crop yield is influenced by several factors, including the frequency and duration of the waterlogging event(s) and timing with respect to the growth stage of the crop (Johnston 1999), the sensitivity of the species or variety (Brisson et al. 2002; Celedonio et al. 2014; Ghobadi et al. 2016), and soil temperatures during the flooding event (Trought and Drew 1980). Higher floodwater temperatures exacerbate flooding damage (Fausey and Mcdonald 1985), and oxygen depletion in waterlogged soil occurs rapidly across most temperature ranges (Trought and Drew 1980). Waterlogging is particularly detrimental to crop yields when it occurs during germination or the early vegetative stages (Cannell et al. 1980, 1984, 1985). Consequently, crop responses to water stress vary depending on both internal growth characteristics and external environmental factors.

Waterlogging‐tolerant plants exhibit various morphological and physiological adaptations to cope with stress. One key mechanism is the formation of adventitious roots, which develop from non‐root tissue to enhance oxygen uptake (Steffens and Rasmussen 2016). Additional adaptations include the rapid elongation of apical meristematic tissue, the development of barriers to radial oxygen loss (ROL), and the formation of air films on the upper cuticle, all of which improve gas exchange and reduce oxygen loss under waterlogged conditions (Hattori et al. 2009; Pedersen et al. 2009; Yamauchi et al. 2018; Qi et al. 2019). Another critical response is the activation of fermentative metabolism to sustain ATP production during low oxygen availability. This involves increased activity of alcohol dehydrogenase (ADH) and the upregulation of ADH1 expression in Arabidopsis (Sun et al. 2018). However, flooding stress also induces the accumulation of harmful compounds, such as reactive oxygen species (ROS) and malondialdehyde (MDA) (Jia et al. 2019). To mitigate this damage, plants activate protective mechanisms, including the production of proline and glutathione, along with the activation of antioxidant enzymes. These responses help neutralize ROS and maintain cellular integrity (Ye et al. 2015; Wang et al. 2019).

Numerous studies have shown that teff is tolerant to short seasonal periods (for a week or two) of waterlogging in Ethiopia (Teklu and Tefera 2005; Minten et al. 2013; Assefa et al. 2015), as it has the ability to withstand anaerobic conditions better than many other cereals (Ketema 1997). A recent study by Cannarozzi et al. (2018) reported variation in waterlogging tolerance among teff varieties in the early and tillering stages. However, to the best of our knowledge, no research has been carried out to evaluate the effect of long‐duration waterlogging on the different yield components of teff. In any case, most of the previous studies were conducted under the application of N and P fertilizers, assuming other nutrients were supplied from the soil.

The objective of this study was to evaluate five waterlogging durations and to quantify the effect on teff growth, yield, nutrient uptake, and partitioning. Though this was a one‐season pot experiment that may not exhaustively address issues related to the effect of waterlogging on teff, the information generated from this study would help initiate further detailed studies. The findings from this study may be valuable in the development of an improved management practice to optimize teff production under a changing climate.

## 2 Materials and Methods

**FIGURE 1:**
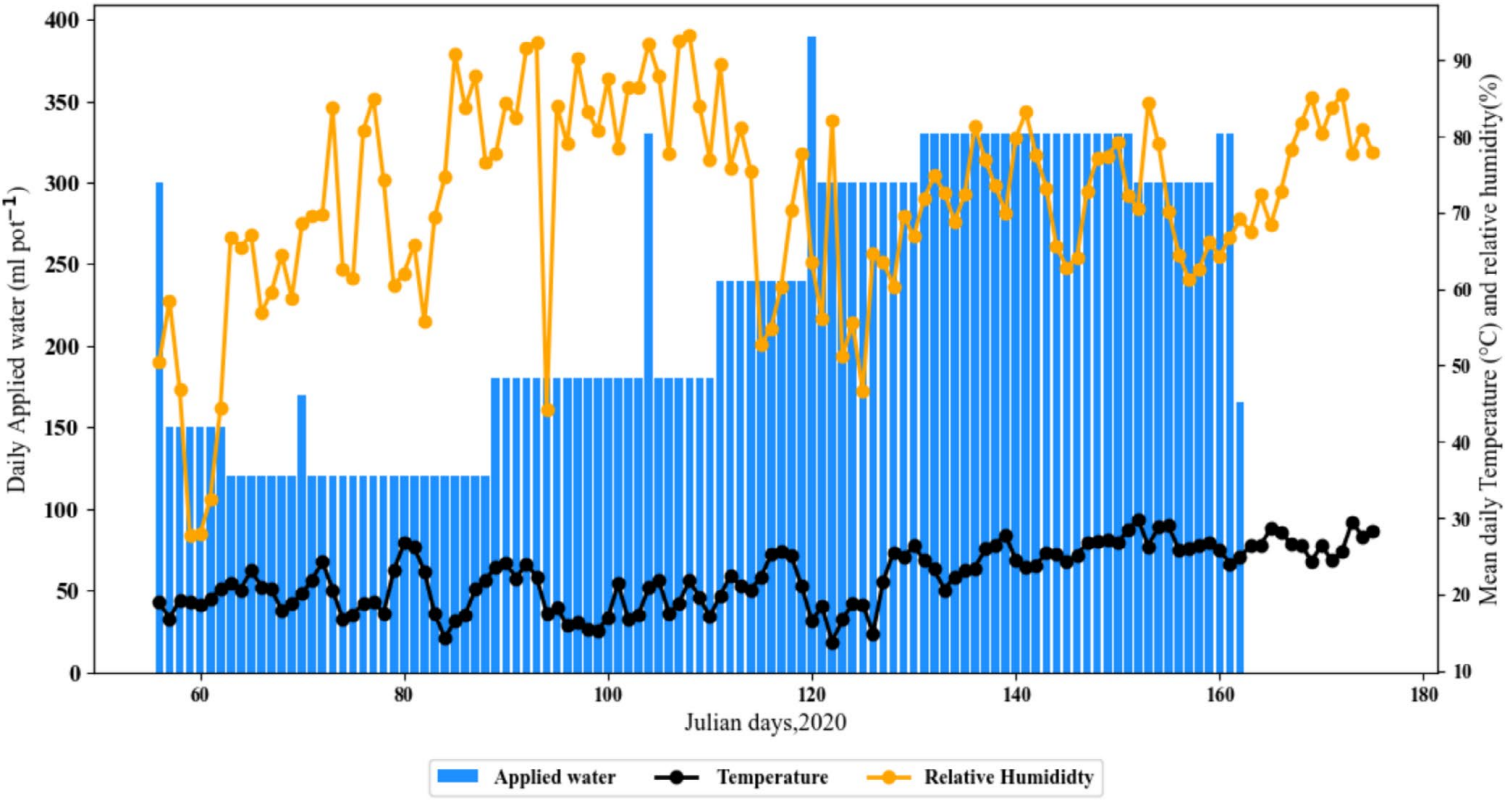
Amount of water applied pot^−1^, mean daily air temperature (°C), and mean daily relative humidity (%) at IFDC Greenhouse, 2020.

A pot experiment was conducted between March and July 2020 at the International Fertilizer Development Center (IFDC) greenhouse at Muscle Shoals, Alabama, USA (34.76929° N, 87.654524° W) at an altitude of 167 m above mean sea level. Data on daily temperatures and relative humidity were recorded every 15 min using an automated weather station, and the daily averages of these parameters and the amount of water applied daily per pot were reported (Figure 1). The soil was Sumter clay loam (fine‐silty, carbonatic, thermic Rendollic Eutrudepts) (USDA 2014). The Sumter series consists of moderately deep (> 152.4 cm), well‐ or moderately well‐drained, slowly permeable soils with medium to rapid runoff. It typically has dark grayish, clayey surface layers over clayey subsoil. It has a bulk density of 1.2–1.6 g cm^−3^ and a permeability of 15.24–50.8 mm/h. The soil also had sand, clay, and silt content of 39.9%, 30.1%, and 3.0%, respectively. Soil samples were analyzed for selected chemical properties using standard procedures for each (Table 1). Uniform rates of N as urea, potassium (K) as muriate of potash (KCl), phosphorus (P) as monocalcium phosphate (MCP), and micronutrients were applied at 200, 100, 150, 24, 2, 6, 3, 2, 0.5, and 0.05 mg kg^−1^ of N, P, K, magnesium (Mg), copper (Cu), zinc (Zn), iron (Fe), manganese (Mn), boron (B), and molybdenum (Mo), respectively. These nutrient rates were selected based on the previous study in the same greenhouse for the same soil and for the same teff variety (result not published). All fertilizers were applied basally, except N, which was applied in three splits (one‐third at transplanting, 20DAT, and 40DAT).

**TABLE 1:** Selected soil chemical characteristics and the analysis method used.

Soil parameter	Extraction method	Unit	Value	Reference
pH	1:2 H_2_O		8.26	Thomas (1996)
Zinc	DTPA	mg kg^−1^	0.23	Lindsay and Norvell (1978)
Extractable P	Fe‐oxide Pi	mg kg^−1^	8.27	Menon et al. (1989)
Sulfate‐S	MCP	mg kg^−1^	5.26	Fox et al. (1964)
NH_4_‐N	KCl	mg kg^−1^	9.2	Keeney (1982)
NO_3_‐N			13.59	
Exchangeable potassium	NH_4_Cl	cmol kg^−1^	0.26	Sumner (1996)
Organic carbon	Walkley and Black	g kg^−1^	18.8	Walkley and Black (1934)

Pots with an eight‐kilogram capacity were used for the study. Soil and the predetermined fertilizer were thoroughly mixed in a five‐gallon bucket using an electric drum mixer before transferring to the experimental pots. Prior to sowing, the soil pots were treated with a fungicide: 1 L Actinovate solution (2.8 g per gallon of water). A high‐yielding and white‐seeded teff variety, Quncho (DZ‐Cr‐387 RIL‐355) which was sourced from a breeder and researcher in Phoenix, Arizona, USA, was used as the test crop. Seeds were sown in a seedling tray with growing media and allowed to grow for 2 weeks after emergence. Then, two seedlings were transplanted into each pot and were respectively subjected to five durations of waterlogging, according to treatment. Waterlogging for all treatments but the control started 1 week after transplanting. Throughout the waterlogging durations, the water level was consistently maintained at about 3 cm. The five durations of waterlogging and their descriptions are presented in Table 2. The pots used for the waterlogging experiment were lined with plastic, and no water leaked from the pots. After each treatment duration, the plastic lining was cut at the bottom, and holes were opened to drain the water. The drained water was collected with the pot cover and was added back again for irrigating the plants. For the pots with 0 days of waterlogging and those drained after their duration had been completed, deionized water was applied using an automatic drip irrigation system, and the soil moisture was kept at approximately 80% field capacity. The pots were kept weed‐free for the entire growing period.

**TABLE 2:** Treatments and their descriptions.

No.	Treatment	Description
1	0DAT (control)	The pots were not waterlogged throughout the growing period. Optimum moisture was maintained
2	20DAT	The pots were waterlogged for 20 days and then drained. Optimum moisture was maintained after drainage
3	40DAT	The pots were waterlogged for 40 days and then drained. Optimum moisture was maintained after drainage
4	60DAT	The pots were waterlogged for 60 days and then drained. Optimum moisture was maintained after drainage
5	WHOLE	The pots were waterlogged for the entire growth season

The experiment was laid out in a complete randomized design (CRD) with three replicates. Observations were recorded on the growth performance of teff at different stages. Accordingly, the number of tillers per plant was counted three times in 20‐day intervals from April 29 to June 9, 2020, and at harvest. Time of heading and number of heads emerged at 2–3‐day intervals were also recorded. At maturity, the crops were manually harvested from the ground from each pot. At the time of harvest, plant height, panicle length, and number of primary panicle branches were recorded. The plant parts were divided into roots, straw, and grain. After the straw and grain samples were dried to a constant weight in an oven at a temperature of 100°C, they were threshed and cleaned to determine grain yield and straw yield. Similarly, the roots were separated from the soil, washed with distilled water, and dried at 100°C to a constant weight to determine root dry weight. The dried samples were milled, and the root, straw, and grain were analyzed for nutrient content using the following procedures. N was determined using a micro‐Kjeldahl method, a modification of the aluminum block digestion technique described by (Gallaher et al. 1975). P was analyzed using the molybdenum blue method (Murphy and Riley 1962). K was analyzed using a Spectro Arcos ICP‐OES analyzer (SPECTRO Analytical Instruments GmbH, Kleve, Germany). The quantities of nutrients in the plant tissues were expressed as milligrams of nutrient per kilogram of plant on a dry weight basis. Nutrient uptake was calculated as a product of nutrient concentration and dry weight of the respective plant parts and expressed as milligrams per pot.

Analysis of variance (ANOVA) was performed using R (R Core Team 2023). Tukey's procedure was performed to determine significant differences between individual means at *p* < 0.05.

## 3 Results

### 3.1 Soil Properties

Selected chemical properties of the soil used for the study are provided in Table 1. Analysis results showed that the soil used for the study was slightly alkaline and had essential nutrients in varying concentrations; P, K, and Zn were under the low (deficient) category and S was under the medium category according to ratings by Marx and Hart (1999).

### 3.2 Yield and Yield Components

Generally, waterlogging duration did not influence the phenological development of teff; no significant timing differences were observed in the tillering, flowering, and maturity stages between the waterlogged plants and the non‐waterlogged (0DAT) plants. Overall, teff exhibited tolerance to waterlogging in this experiment. There was no significant difference between treatments for grain yield, number of fertile tillers per plant, panicle‐related parameters, and plant height (Table 3). However, there was a significant (*p* = 0.005) difference between waterlogging duration treatments for aboveground biomass and straw yield. Similarly, harvest index and root weight were significantly (*p* = 0.03) affected by waterlogging duration (Table 3).

**TABLE 3:** Effect of waterlogging on yield and yield components of teff. Data shown are means from 3 replications.

Treatment	AGB yield	Grain yield	Straw yield	HI	Fertile tillers/plant	Panicle weight	Grain weight/panicle	Plant height	Panicle length	Root weight
	gram pot^−1^			%		gram		cm		gram
0DAT	210 ± 14.0^a^	37 ± 2.6	173 ± 13.7^ab^	18 ± 0.01^ab^	44	0.90	0.46	183	65.8	17 ± 17.0^b^
20DAT	219 ± 17.1^a^	33 ± 5.9	189 ± 15.6^a^	14 ± 0.03^b^	31	1.04	0.51	209	63.0	21 ± 4.6^b^
40DAT	190 ± 16.1^ab^	32 ± 1.8	158 ± 14.7^ab^	17 ± 0.01^ab^	29	1.23	0.63	225	71.8	16 ± 4.5^b^
60DAT	227 ± 6.8^a^	38 ± 5.4	188 ± 11.2^a^	18 ± 0.03^ab^	39	0.92	0.51	208	63.3	26 ± 33.0^b^
WHOLE	172 ± 14.9^b^	33 ± 2.6	139 ± 13.2^b^	24 ± 0.05^a^	30	0.99	0.68	197	60.1	46 ± 136.3^a^
Pr > F	0.005	0.14	0.005	0.03	0.13	0.68	0.57	0.11	0.11	0.03

*Note:* Means ± standard deviations followed by the same letter within a column are not significantly different at *p* < 0.05.

Abbreviations: AGB, aboveground biomass; DAT, days after transplanting; HI, harvest index.

### 3.3 Time to Tillering and Heading

**FIGURE 2:**
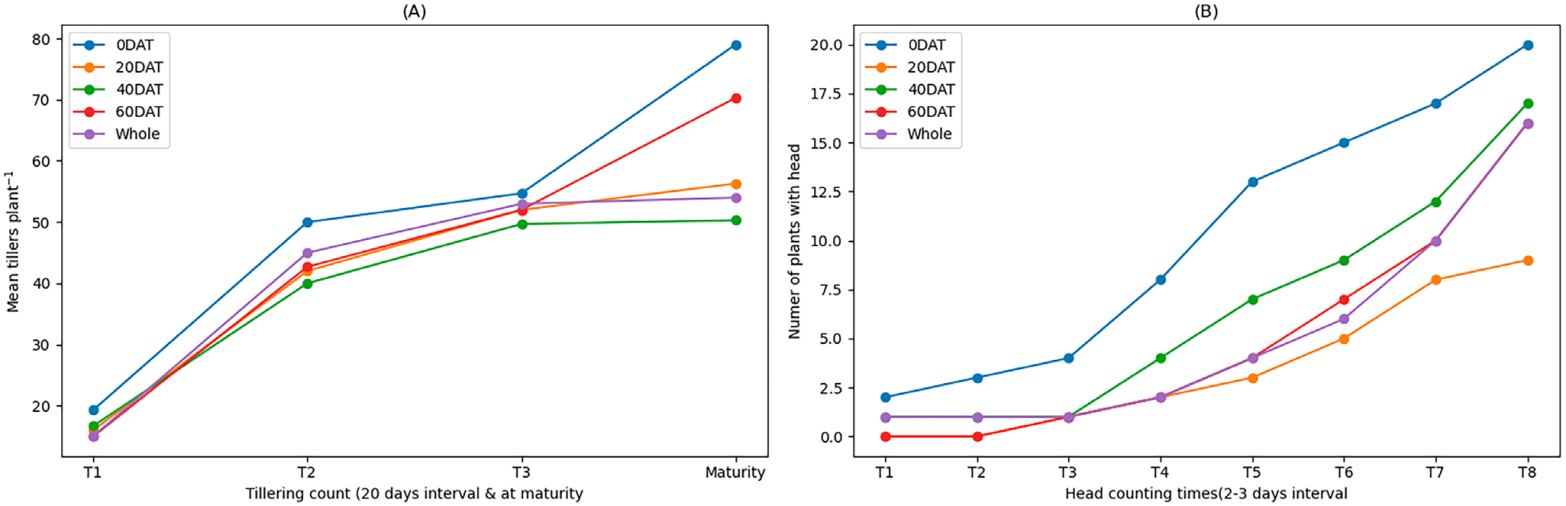
Variations in tiller number at 20‐day intervals (A) and number of heads emergence counted at 3‐day intervals (B) counted to see if there is variation in tillering and heading time among treatments. T1, T2… show the time of sampling.

Though plants started heading at the same time, the number of heads that had emerged at each specific time was different (Figure 2). However, the difference was not statistically significant.

### 3.4 Total Number of Tillers and Fertile Tillers per Plant at Maturity

In this experiment, the number of tillers per plant varied with waterlogging duration. The average fertile tillers per plant at maturity ranged from 30 to 39. In general, waterlogging duration did not have much influence on tillering time, but it did reduce the number of fertile tillers per plant (Figure 2; Table 3). The control treatment (0DAT) produced a higher percentage (2%–23%) of tillers that developed into grain‐bearing heads than the waterlogged treatments (Table 3).

### 3.5 Plant Height, Panicle Length, Panicle Weight, and Number of Panicle Branches

Substantial differences in plant height were observed among the different waterlogging durations (Table 3). The different waterlogged treatments produced an 8%–23% increase in plant height over control treatment. Similarly, panicle length showed variation among treatments, but the difference was not significant (*p* < 0.114). Waterlogging did not significantly (*p* < 0.478) affect the number of panicle branches. A higher number of panicle branches was recorded for the 40DAT followed by 20DAT treatment, and the lowest for the control treatment, although the difference was not significant (*p* < 0.478) (Table 3). Panicle weight, grain weight/panicle, plant height, and panicle length yield showed an increasing trend until 40DAT and decreased afterward.

### 3.6 Straw Yield and Root Dry Weight

**FIGURE 3:**
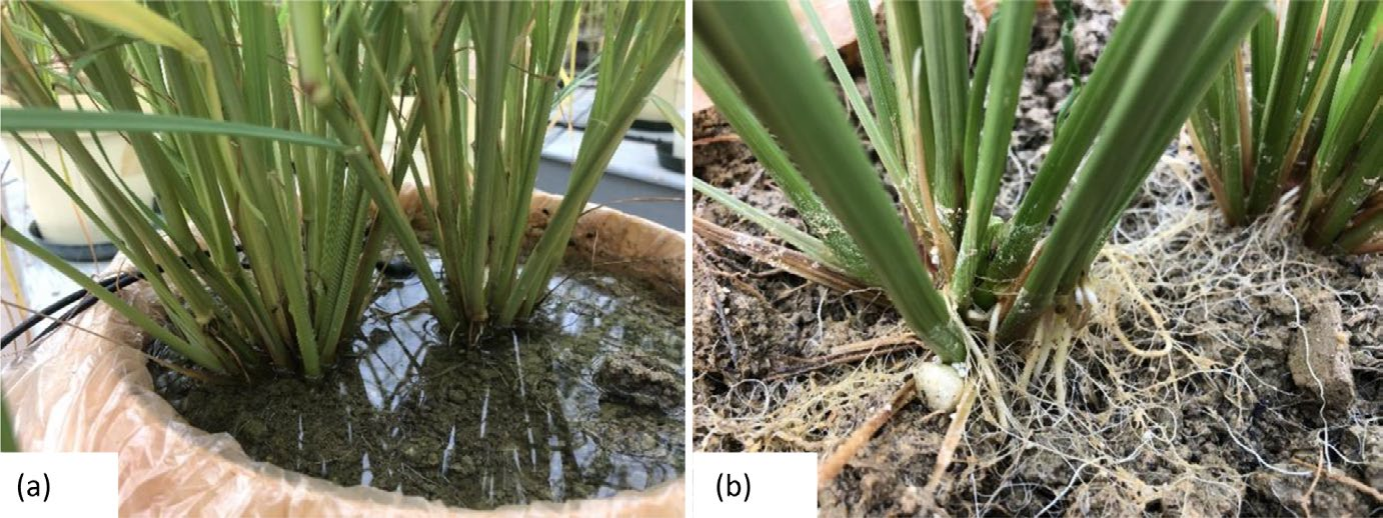
A photograph showing root growth of waterlogged teff (a) 68DAT and (b) drained teff (40DAT).

Figure 3a,b shows the performance of teff under waterlogged conditions and immediately after draining, where strong stems and roots appeared on the surface of the soil respectively. In this study, straw dry weight showed an inconsistent trend (Table 3); it was lower than the control for the 40DAT and the entire growth period treatments by 8% and 20%, respectively, and higher than the control for the 20DAT and 60DAT treatments by 9% each.

The root dry weight values demonstrated that, as the duration of waterlogging increased, root weight also increased. Accordingly, the treatment with waterlogging for the entire growth period had significantly (*p* < 0.03) greater root weight than the treatment without waterlogging (Table 3).

### 3.7 Grain Yield and Harvest Index

Grain yield is the priority for teff‐growing farmers and was the main target of our investigation. Grain yield varied among treatments but did not show a clear trend with increased waterlogging duration. Grain yield for the 20DAT, 40DAT, and entire growth period treatments was lower than the control yield by 19%, 15%, and 11%, respectively, and the 60DAT treatment showed a 3% increase. The main reason contributing to the difference in grain yield came from variations in the number of fertile panicles and the length of the panicles, which is directly proportional to the number of grains per panicle. However, no significant (*p* < 0.137) differences between waterlogging treatments were found (Table 3). The mean panicle weight and grain yield were not significantly (*p* = 0.68, *p* = 0.14, respectively) affected by variations in waterlogging duration, confirming that waterlogging decreases grain yield per plant by decreasing kernel number per plant (Celedonio et al. 2014).

### 3.8 Total Aboveground Biomass Yield

The total aboveground biomass yield (AGBY), i.e., grain yield plus straw yield, obtained was significantly(*p* < 0.005) higher for the 60DAT treatment, followed by 20DAT, while the lowest was from the treatment waterlogged for the entire season (Table 3). It follows the same trend as straw yield, and there was an increase of 4% and 8% for the 20DAT and 60DAT treatments, respectively, over the control, while a reduction of 10% and 18% was observed with the 40DAT and entire growing season treatments, respectively.

### 3.9 Nutrient Uptake by Roots, Straw and Grain

The results of our experiment indicated that waterlogging duration significantly affected uptake of N (*p* = 0.004), P (*p* = 0.002), and K (*p* = 0.005) in roots, P (*p* = 0.001), and K (*p* = 0.002) uptake in straw and N (*p* = 0.03), and P (*p* = 0.04) uptake in grains of teff (Table 4). The uptake of N, P, and K showed a general increasing trend with increasing waterlogging duration in teff roots. On the other hand, in both grain and straw, the uptake of N showed a decreasing trend until 40DAT and increased again. The uptake of P increased with increasing waterlogging duration until 60DAT and decreased afterward. Uptake of K showed different trends in grain and straw, where it increased until 60DAT in straw, whereas it decreased with increasing waterlogging duration. The uptake of N, P, and K was lowest in roots compared to straw and grain.

**TABLE 4:** Effect of waterlogging on N, P, and K uptake by teff root, straw, and grain.

Treatment	Root uptake (mg/pot)			Straw uptake (mg/pot)			Grain uptake (mg/pot)		
	N	P	K	N	P	K	N	P	K
0DAT	86 ± 17.0^b^	5.1 ± 0.9^b^	15 ± 2.3^b^	558 ± 67.9	111 ± 9.4^a^	1233 ± 60.3^bc^	553 ± 66.1^a^	159 ± 10.2a	238 ± 28.1
20DAT	112 ± 4.6^b^	8.1 ± 0.8^b^	18 ± 2.1^b^	504 ± 94.1	129 ± 24.6^a^	1325 ± 201.7^abc^	433 ± 77.8^ab^	133 ± 29.5a	192 ± 47.4
40DAT	74 ± 4.5^b^	4.7 ± 0.7^b^	14 ± 1.4^b^	420 ± 43.8	136 ± 7.9^a^	1472 ± 62.2^ab^	425 ± 11.8^ab^	147 ± 1.1ba	211 ± 10.7
60DAT	104 ± 33.0^b^	7.8 ± 2.9^b^	20 ± 7.7^b^	466 ± 58.5	132 ± 7.6^a^	1510 ± 27.7^a^	516 ± 67.8^ab^	161 ± 15.7a	241 ± 30.6
WHOLE	320 ± 136.3^a^	29.4 ± 12.7^a^	48 ± 18.9^a^	487 ± 82.3	70.7 ± 2.9^b^	1059 ± 36.8^c^	398 ± 25.0^b^	120 ± 8.1b	208 ± 14.2
Pr > F	0.004	0.002	0.005	0.28	0.001	0.002	0.038	0.048	0.28

*Note:* Means ± standard deviation followed by the same letter within a column are not significantly different at *p* < 0.05.

Root N uptake in the 0DAT treatment was lower than that in the other treatments except 40DAT. However, in the same treatment, the N uptake in grain yield and straw was higher than that in the other treatments.

## 4 Discussion

### 4.1 Growth, Yield, and Yield Components

The effect of waterlogging duration on yield and yield components of teff showed variable results. Its influence on the phenological development of teff and timing differences in the tillering, flowering, and maturity stages between the waterlogged plants and the non‐waterlogged (0DAT) plants was not significantly different. Similar findings were reported for barley (Pang et al. 2004) and wheat (Malik et al. 2001, 2002) where the number of tillers per seedling decreased after 3 weeks of waterlogging and during tillering of wheat, respectively, compared to plants grown in well‐drained soil. This reduction may be attributed to the application of balanced fertilization in addition to the inherent adaptive mechanisms of the crop. Several studies have suggested that exogenous application of fertilizers could be effective in helping plants recover from waterlogging injury (Ashraf et al. 2011; Habibzadeh et al. 2012; Najeeb et al. 2015; Noreen et al. 2018). Previous research reports have indicated that N fertilizer application has beneficial effects on improving yields of maize (Meyer et al. 1987), wheat (Robertson et al. 2009; Wu et al. 2014), and cotton (Zhou and Oosterhuis 2012) under waterlogging conditions. Nielsen (2019) indicated that waterlogging can increase N losses via denitrification, and hence, the addition of N fertilizer may accelerate plant adaptive mechanisms to waterlogging, such as root regrowth after flooding and adventitious root growth. Similarly, Swarup (1993) reported a significant increase in wheat grain yield on flooded sodic soils due to increased rates of top‐dressed urea.

Application of K has increased teff yield and yield components in waterlogged Vertisols (Habte and Boke 2017; Misskire et al. 2019; Mulugeta et al. 2020). On the other hand, Ashraf et al. (2011) also indicated that K supplementation increased plant growth, photosynthetic pigments, and photosynthetic capacity in waterlogged cotton. Applications of P and B have also improved crop yields of barley (Ylivainio et al. 2018) and maize (Sayed 1998), respectively, grown under waterlogging conditions. Various researchers have reported that the effects of waterlogging were diminished due to application of fertilizer on barley (Pang et al. 2007), wheat (Kaur et al. 2017; Pereira et al. 2017; Zheng et al. 2017), maize (Kaur et al. 2018; Rao et al. 2002), and canola (Habibzadeh et al. 2012).

Teff behaves similarly to rice in waterlogging tolerance in this experiment. Previous results showed that in a waterlogging‐tolerant rice, no difference in time to flowering was observed under continuous waterlogging compared to non‐waterlogging conditions (Stuerz et al. 2014). On the contrary, exposure of wheat and barley cultivars to waterlogging during the early stages of development (leaf 1 to leaf 7 in the main stem) significantly delayed flowering time, with barley being the most affected (De San Celedonio et al. 2016).

However, there were substantial differences in plant height among the different waterlogging durations (Table 3), which produced an 8%–23% increase in plant height over control treatment. Similar results were reported by Polthanee et al. (2008) on kenaf, who reported that the plant height was higher by 108% and 107% over the control in the early and mid‐season flooded plants. Panozzo et al. (2019) and Surukite et al. (2020) also found that there was a general increase in plant height in some of the maize hybrids and on jungle flame, respectively, subjected to waterlogging. The increase in height may be one of the physiological responses of teff to waterlogging. Previous reports have indicated that adventitious rooting, increases in plant height, and the resulting increase in the proportion of aboveground biomass were some of the morphological‐level responses to waterlogging (Grimoldi et al. 1999; Naidoo and Mundree 1993). Morphological responses of crops to waterlogging vary across cultivars, and taller plant height is probably due to better oxygen diffusion in the shoot tissues of these hybrids than in others (Armstrong and Brändle 1994). Similarly, the increase in the number of panicle branches might have contributed to higher panicle weights in these two treatments over others that have longer panicles but less weight, panicle weight being a combination of panicle branches, height, and seed weight. This was in line with previous reports on rice where panicle weights of rice landraces were positively correlated with panicle number, grain number, and leaf area and negatively associated with panicle length, panicle angle, and chaff number (Panda et al. 2020).

Panicle weight, grain weight/panicle, plant height, and panicle length yield showed an increasing trend until 40DAT and decreased afterward. This might be due to the preference to have more tillers over the main stem, a morphological change caused by a shift in the allocation priority of photosynthetic products as a plant adaptive response to waterlogging conditions (Maai 2024). Though more tillers were produced, the number of fertile tillers was lower as the waterlogging duration increased, which may contribute to lower panicle and grain weight per plant. Similar results were reported by Zhang et al. (2023).

Previous studies show that, depending on the crop, the response of straw dry weight to waterlogging varies. Waterlogging treatment significantly increased shoot dry weight in tall fescue and Kentucky bluegrass ( *Poa pratensis* ) (Zhang et al. 2013). Similarly, in a study that investigated 12 *Brachiaria humidicola* germplasm accessions, it was found that some increased their shoot dry mass, whereas others showed a reduction in shoot dry mass under waterlogging conditions (Cardoso et al. 2013, 2014). For teff, the increase in straw weight could be associated with the increase in plant height, tiller number, or a combination of the two, depending on the duration‐specific effects.

In waterlogging‐tolerant crop species, one of the most important characteristics for growth and grain production is root adaptation because the deficiency of oxygen around the root zone is the main cause of damage under waterlogging stress. In our experiment, we found that adventitious roots emerged from the stems above the water line on the treatments with continuous waterlogging. This was visibly observed while the pots were drained (Figure 3b). Some of these roots floated in the water and did not penetrate the soil. This could be a mechanism to adapt to the waterlogging condition as many plant species produce adventitious roots during waterlogging, with some emerging into the soil and others along the soil surface. During deeper floods, some even grow into the water column (Visser and Voesenek 2004); these are referred to as aquatic adventitious roots. An increased proportion of lateral roots in the upper layers is thought to be of adaptive value by compensating for the reduction of absorptive root surface (Cardoso et al. 2014).

In our experiment, root dry weight demonstrated that, as the duration of waterlogging increased, root weight also increased. This result is consistent with that of Matsuura et al. (2016), who studied the effect of waterlogging on four millet varieties and found that the plant with the best waterlogging tolerance among the four millet varieties, *P. sumatrense* , increased the number of its crown roots and root biomass under waterlogging. A study on forage grass, *Brachiaria humidicola* , also showed that waterlogging increased the proportion of lateral roots in the upper layers of the soil (Cardoso et al. 2014). Another experiment on soybean reported higher root dry weight under hypoxic conditions than under aerobic conditions, which indicates the potential tolerance of the cultivar to hypoxia (Jitsuyama 2015). Waterlogging‐tolerant crop varieties showed a better ability to develop more adventitious roots and a larger percentage of aerenchyma to take up K^+^ in the mature root zone (Pang et al. 2004), to maintain higher O_2_ uptake in the mature root zone (Pang et al. 2006), and to tolerate secondary metabolites associated with waterlogged soil conditions (Pang et al. 2007). In a sugarcane experiment in India, total root weight increased by 4.02% in waterlogging conditions in comparison to normally grown (unstressed) canes (Misra et al. 2020). Zhai et al. (2013) also showed that maize exposed to waterlogging stress formed more crown roots than the control and that the waterlogging‐tolerant line possessed more crown roots than the waterlogging‐sensitive line.

More roots developed in waterlogged plants, suggesting that teff might have utilized aerenchyma formation in adventitious roots as a mechanism to tolerate waterlogging. An increased number of newly emerged adventitious roots can compensate, at least partially, for the growth inhibition or even death of distal portions of roots present when waterlogging occurs (Jackson 1984). The application of balanced fertilizer could have also contributed to better root development.

The effect of waterlogging duration on grain yield and harvest index was also evaluated. The results indicated that though grain yield varied among treatments, it did not show a clear trend with increased waterlogging duration. The main reason contributing to the difference in grain yield came from variations in the number of fertile panicles and the length of the panicles, which is directly proportional to the number of grains per panicle. However, no significant (*p* < 0.137) differences between waterlogging treatments were found.

In all cereals, kernel number per plant is a crucial yield determinant, as it is the component most strongly related to grain yield and is also more variable in response to environmental and stress conditions (Mahadevan et al. 2016). In wheat, waterlogging tolerance and biomass production are highly dependent on the environment (Setter et al. 2009), and it is hypothesized that the high rates of yield loss due to soil waterlogging are related to the relatively high temperatures that accompany the excess water (Musgrave and Ding 1998). Qian et al. (2018) reported a greater effect on cotton yield from the interaction of high temperature and waterlogging than that of high temperature and waterlogging alone. However, for rice, the interaction of temperature and waterlogging had less effect on chlorophyll and soluble sugar than temperature or water stress alone (Zhen et al. 2019) and high temperature and waterlogging increased the leaf area and dry matter accumulation of the shoot (Zhen et al. 2019, 2020). This is because rice is a wetland crop that has a stronger ability to adapt to an anoxic environment than cotton. Similar conditions might also apply for teff, as it also has relatively better adaptation to waterlogging. In our research, during the period of waterlogging from 90DAT to harvest (entire growth period), the mean temperatures were between 25°C and 31°C (Figure 1), which is at the upper limit for teff growth. This may have also influenced grain yield, especially for the longer duration of waterlogging treatments.

### 4.2 Nutrient Uptake and Partitioning

The results of our experiment indicated that waterlogging duration significantly affected nutrient uptake in different parts of the teff plant. The uptake of N, P, and K showed a general increasing trend with increasing waterlogging duration in teff roots. This might be due to the formation of more adventitious roots as a tolerance mechanism that helps the roots take more nutrients (Wiengweera and Greenway 2004; Steffens et al. 2013; Ayi et al. 2016). On the other hand, the resulting decreasing trend in N uptake until 40 DAT increased again, in both grain and straw, which could be due to the application of N at split and higher rates (200 mg kg^−1^) that improved nutrient uptake efficiency (Aerts et al. 1991). Besides this, it might also be due to high nutrient demand under soil anoxia as waterlogging‐tolerant plants because plants are able to maintain or even increase their biomass production and nutrient uptake efficiency during soil anoxia (Rubio et al. 1995). The N uptake in the 0DAT treatment exhibited a distinct trend compared to other treatments, particularly evident in the belowground and aboveground parts. Notably, the root N uptake in the 0DAT treatment was lower, whereas the N uptake in straw and grain was higher compared to other treatments. This observed discrepancy may be attributed to the process of N remobilization (Bly and Woodard 2003). Additionally, the application of a higher amount of N fertilizer, coupled with split application strategies, could have influenced the N uptake pattern. Better N management practices often result in improved N absorption efficiency, particularly noticeable at crop maturity (Zheng et al. 2017).

Plants adapt to waterlogging stress by enhanced anaerobic respiration due to lower oxygen diffusion in water. In our experiment, the uptake of N and P showed comparable results in grain and straw, while the uptake of K was higher in straw than in both grain and root. Nutrient uptake by grain was reduced due to waterlogging between 7% and 28% for N, 8% and 25% for P, and 11% and 19% for K compared with non‐waterlogged (drained) plants. Nutrient uptake partitioning was evaluated in root and aboveground (straw and grain) parts, taking the sum of root, straw, and grain as the total. Accordingly, the uptake of N ranged from 7% to 11% in roots, 28% to 35% in straw, and 60% to 68% in grain. The P uptake was also significantly different across the plant parts, ranging from 2% to 13% in roots, 32% to 48% in straw, and 49% to 58% in grain. However, most of the K uptake was by straw, followed by grain. K uptake ranged from 1% to 4% in roots, 81% to 87% in straw, and 12% to 16% in grain. The low concentration of N, P, and K in roots may be due to anoxia, a direct consequence of the low H^+^ ATPase activity (Steffens et al. 2005). Similar results were reported in previous research that indicated that waterlogging stress caused energy shortage, disturbed root hydraulic conductance, reduced nutrient uptake, and decreased photosynthesis, leading to significant yield losses in barley, wheat, and maize (Parent et al. 2008).

## 5 Conclusions

Our results showed that teff can tolerate longer periods of waterlogging with no significant reduction in yield or yield components. Waterlogging reduced the number of tillers forming heads by 2%–23%. Straw and grain yield showed an inconsistent response, with a decrease of 8%–20% and 11%–19%, respectively, in three of the four waterlogging durations, and an increase of 9% and 3%, respectively, in the 60 DAT treatment. Plants have developed numerous adaptive responses that allow them to survive different types of floods and oxygen impedances. The development of adventitious roots and aerenchyma could be the mechanisms that teff uses to tolerate waterlogging, as these may allow it to maintain greater access to oxygen and therefore maintain more normal metabolic processes. The result of this experiment would support the effort to grow teff as a climate‐resilient crop, especially on waterlogged Vertisols, and to the prospects of developing improved germplasm for waterlogging tolerance. Therefore, understanding the regulation of root metabolism and physiological changes during waterlogging conditions, evaluating whether varietal differences in waterlogging tolerance exist, and identifying the physiological mechanisms that help teff tolerate waterlogging are key research areas to be examined. Besides, understanding micronutrient uptake by teff under waterlogging is another area of future research, especially with respect to human and animal nutrition.

## Disclosure

Confirmation on research involving plants: We confirm that this study was in accordance with relevant institutional, national, and international guidelines and legislation.

## Conflicts of Interest

The authors declare no conflicts of interest.

## Data Availability

All the data used for this study are available at https://ifdc.sharepoint.com/:x:/s/SOILSconsortium/EQjshap5oP9GvtvIn2DuBlUBVr6hhkufmnjJ35B3X590zw?e=8SQRPL.
